# Expression of *M*. *tuberculosis*-induced suppressor of cytokine signaling (SOCS) 1, SOCS3, FoxP3 and secretion of IL-6 associates with differing clinical severity of tuberculosis

**DOI:** 10.1186/1471-2334-13-13

**Published:** 2013-01-15

**Authors:** Kiran I Masood, Martin E Rottenberg, Naseem Salahuddin, Muhammad Irfan, Nisar Rao, Berit Carow, Muniba Islam, Rabia Hussain, Zahra Hasan

**Affiliations:** 1 Department of Pathology and Microbiology, Aga Khan University, P.O. Box 3500, Stadium Road, Karachi, 74800, Pakistan; 2 Department of Microbiology and Tumor and Cell Biology, Karolinska Institutet, Stockholm, Sweden; 3 Indus Hospital, Karachi, Pakistan; 4 OJHA Institute of Chest Diseases, DOW University of Health Sciences, Karachi, Pakistan

**Keywords:** SOCS molecules, Cytokine regulation, Tuberculosis

## Abstract

**Background:**

Appropriate immune activation of T cells and macrophages is central for the control of *Mycobacterium tuberculosis* infections. IFN-γ stimulated responses are lowered in tuberculosis (TB), while expression of Suppressor of Cytokine Signaling (SOCS) molecules – 1 and 3 and CD4^+^CD25^+^FoxP3^+^T regulatory cells is increased. Here we investigated the association of these molecules in regard to clinical severity of TB.

**Methods:**

Peripheral blood mononuclear cells (PBMCs) were isolated from patients with pulmonary TB (PTB, n = 33), extra-pulmonary TB (ETB, n = 33) and healthy endemic controls (EC, n = 15). Cases were classified as moderately advanced or far advanced PTB, and less severe or severe disseminated ETB. *M*. *tuberculosis* -stimulated IFN-γ, SOCS1, SOCS3 and FoxP3 gene expression and secretion of Th1 and Th2 cytokines was measured. Statistical analysis was performed using Mann–Whitney U, Wilcoxon Rank and Kruskal Wallis non-parametric tests.

**Results:**

In un-stimulated PBMCs, IL-6 (p = 0.018) and IL-10 (p = 0.013) secretion levels were increased in PTB while IL-10 was also increased in ETB (p = 0.003), all in comparison with EC. *M*. *tuberculosis*-stimulated IL-6 (p = 0.003) was lowered in ETB as compared with EC. SOCS1 mRNA expression in *M*. *tuberculosis* stimulated PBMCs levels in moderately advanced PTB (p = 0.022), far advanced (p = 0.014) PTB, and severe ETB (p = 0.009) were raised as compared with EC. On the other hand, SOCS1 mRNA titers were reduced in less severe ETB, in comparison with severe ETB (p = 0.027) and far advanced PTB (p = 0.016). SOCS3 mRNA accumulation was reduced in far advanced PTB (p = 0.007) and FoxP3 mRNA expression was increased in less severe ETB as compared with EC (p = 0.017).

**Conclusions:**

The lowered SOCS1 mRNA levels in patients with less severe extra-pulmonary TB as compared to those with more severe ETB and PTB may lead to elevated IFN-γ pathway gene expression in the latter group. As localized ETB has shown to be associated with more effective Th1 immunity and adaptive responses, this suggests a role for SOCS1 in determining disease outcome in extra-pulmonary TB.

## Background

In 2010 the Global Tuberculosis (TB) report indicated 8.8 million incident cases of TB with 1.1 million reported deaths from TB amongst HIV-negative individuals [1]. In Pakistan, the incidence of TB is 231/100,000 annually with pulmonary TB (PTB) accounting for the majority of all TB cases, while extra-pulmonary TB (ETB) contributes up to 18% of all TB cases [2].

Protection against *M*. *tuberculosis* infection is dependent upon the interaction between host T cells and macrophages and is coordinated by cytokines. CD4^+^ T cells play a central role in containment of *M*. *tuberculosis* infection by secreting Interferon-gamma (IFN-γ) [3]. The enhanced susceptibility to mycobacterial infection of IFN-γ knockout mice [4,5], and of patients with genetic defects in IL-12/ IFN-γ pathway [6], provide strong evidence that IFN-γ is required in defense against *M*. *tuberculosis*. Coordinated Tumor necrosis factor alpha (TNF-α), and Interleukin-12 (IL-12) secretion by macrophages and dendritic cells respectively, is required for protection against *M*. *tuberculosis*[7]. It has been observed that IFN-γ responses in patients with TB and further, pulmonary (PTB) patients with far advanced disease are lowered as compared to those with moderately advanced PTB cases [8]. Similarly, patients with disseminated forms of extra-pulmonary (ETB) disease such as miliary disease have defective IFN-γ dependent responses as compared to patients with localized ETB such as, pleural TB [9]. IL-10 is a down-regulatory cytokine which can balance the effect of IFN-γ [10] and a decreasing IFN-γ/IL-10 ratio across the spectrum of TB is associated with progressive disease [11]. Hence, the clinical severity of TB may be determined by the regulatory balance between activating and inhibitory cytokines in the host.

T regulatory cells (Tregs) are a sub-population of CD4 T cells involved in inhibition of T cell responses and ‘FoxP3” is a classical marker for these cells [12]. T regs are known to secrete IL-10 and TGF-β which restrict T effector cell responses [13]. Increase in CD4^+^CD25^+^ FoxP3^+^ cells has been shown to decrease Th1 cell responses in patients with TB [14].

Host gene expression profiling studies have identified changes in genes regulating lymphocyte trafficking, growth and proliferation and immune signaling pathways during *M*. *tuberculosis* infection [15,16]. Expression levels of the Suppressor of Cytokine Signaling (SOCS) molecules are found to be raised in patients with active TB [17] and are thought to play a role in regulation of cytokine secretion and responses [18]. SOCS1 inhibits STAT1 activation and thereby the response to IFN-γ. The importance of SOCS1 is illustrated by the death of SOCS1^-^/^-^ mice within three weeks after birth due to uncontrolled IFN-γ signaling [18]. SOCS3 another member of SOCS family, inhibits STAT3 activation by gp130, a receptor chain of IL-6R family molecules, amongst other receptors. SOCS3 is preferentially expressed in Th2 cells and hampers the differentiation of Th17 cells [19] and also attenuates the anti-inflammatory effects of IL-6 in macrophages [20].

A differential cytokine activation profile has been associated with the varying clinical severity of patients with TB [21-23]. We hypothesized that the expression of SOCS1, SOCS3 and FoxP3 affects both innate and adaptive immune responses in patients with TB. Therefore, we studied the expression of these molecules and of different cytokines across a clinical spectrum of TB patients as compared to that of EC by measuring their levels in both un-stimulated and *M*. *tuberculosis*-stimulated peripheral blood cells.

## Methods

### Subject selection

Sixty six patients with TB were recruited from Aga Khan University and Hospital (AKUH); Ojha Institute for Chest Diseases, DOW University of Health Sciences (DUHS), and Indus Hospital, Karachi using a cross-sectional study design. The study was approved by Ethical Review Committees of the participating organizations and written informed consent was obtained from all participants. Inclusion criteria were: patients with a confirmed diagnosis of TB who had not received anti-tuberculous therapy (ATT); male or female; between 15–65 years of age; unrelated study subjects. Exclusion criteria were: pregnancy; co-morbid conditions (such as HIV infection, diabetes mellitus, chronic renal failure, chronic liver disease or corticosteroid therapy) and patients with relapsed TB.

Patients were classified as PTB and ETB as per WHO guidelines for treatment of TB [24]. All PTB patients were diagnosed by clinical examination, chest X-ray and had a positive sputum acid-fast bacillus (AFB) microscopy and/or AFB culture [25]. Severity of PTB was classified as moderately advanced (PTB-mod) or far advanced (PTB-adv) disease using a modified classification of the National Tuberculosis Association of the USA based on extent of lung tissue involvement [25]. All ETB patients were diagnosed by clinical examination, radiological imaging or a positive histopathological staining result suggestive of granulomatous inflammation of site specific biopsy or FNAC (fine needle aspirate cytology). Severity of ETB was also assessed by WHO guidelines for treatment of TB, according to which cases with tuberculous lymphadenopathy and unilateral pleural effusion were classified as less-severe ETB (L-ETB) and spinal, abdominal, ovarian and bilateral pleural effusion TB were classified as severe disseminated ETB (D-ETB) [24].

Asymptomatic healthy volunteers who were BCG vaccinated staff at AKUH were recruited (EC, n = 15) after tuberculin skin testing (TST). TST was assessed by intradermal administration of five tuberculin units and read after 48 h. An induration of < 10 mm was used as a cut-off for negative responses. Only TST negative EC were selected as the un-infected control group for the study.

The age of TB patients and ECs was comparable (mean ± SD; EC, 30.4 ± 10.1 y; TB, 31 ± 14.9 y) as was their gender distribution (Male/Female; EC, 08/07; TB, 23/43). Characteristics of study subjects with PTB and ETB and the specific disease site for each TB case is listed in Table 1. Total leukocyte (TLC) counts and neutrophils were found to be significantly raised in all TB groups as compared with EC, while lymphocyte counts were reduced in the PTB and ETB groups as compared with EC (Additional file 1: Table S1).

**Table 1:** Characteristics of the study subjects

**Site**	**N**	**Abscess**	**Microscopy**	**Radiology**	**AFBC**	**Histopathology**
**PTB**	33		14/23	30/30	10/16	2/23
**ETB**						
**L-ETB**						
Lymph Node	19	1	0/5	3/13	1/4	16/16
Unilateral Pleural	7		0/3	7/7	1/2	0/1
**D-ETB**						
Spine	4	2	0/2	1/2	1/2	3/3
Abdominal	1		1/1	1/1	0/1	N/A
Ovarian	1		N/A	0/1	1/1	1/1
Bilateral Pleural	1		0/1	1/1	N/A	N/A

PTB, pulmonary TB; ETB, extra-pulmonary TB; L-ETB, less severe ETB; D-ETB, disseminated ETB; N, number of subjects; Microscopy, acid fast bacillus smear using Ziehl Neelsen staining; Radiology, XRay/ CT scan/ or MRI; AFBC, acid fast bacilli culture by MIGIT system (Becton Dickinson, USA); histo-pathological staining of biopsy material and or FNAC (fine needle aspirate cytology) where relevant.

The numbers indicate those diagnosed as positive /of total by each method.

N/A, not available.

### Mycobacterium culture

*M*. *tuberculosis* H37Rv (ATCC) was cultured in a 7H9 Middlebrook medium supplemented with 0.02% glycerol, 10% albumin-dextrose-catalase Middlebrook enrichment, and 0.5% Tween 80 (Difco Laboratories, Detroit, MI) up to logarithmic phase. Aliquots of mycobacteria were frozen in growth medium containing 15% glycerol and were stored at ^_^70°C. For the infection assay, aliquots of mycobacteria were freshly thawed, washed three times in PBS, and diluted to MOI of 2.5. Bacterial viability was greater than 80% in each case. To prevent clumping of the mycobacteria, the cell suspension was sonicated prior to use as described previously [26].

### Infection of peripheral blood mononuclear cells (PBMCs) with *M*. *tuberculosis* H37Rv

Ten ml of venous blood was used to obtain a buffy coat layer containing peripheral blood mononuclear cells (PBMCs) using Ficoll-histopaque density gradient. PBMCs plated were 10^6^ cells per well and were co-incubated with *M*. *tuberculosis* H37Rv (ATCC) culture at MOI-2.5. PBMCs were cultured for 18 hours in RPMI 1640 medium, L-glutamine 2 mM (Sigma Aldrich, USA) with 10% autologous serum at 37°C after which cellular supernatants were collected and total RNA was isolated as described previously [27].

### Real time PCR

Total RNA was isolated from PBMCs using Trizol reagent (Invitrogen, USA). RNA (1 μg) was reverse transcribed using MulV reverse transcriptase (Invitrogen, USA) as described previously [28]. Real time PCR was performed in duplicate 20 μl reactions containing Platinum® SYBR® Green qPCR Supermix-UDG (Invitrogen), 150 nM forward and reverse primers, and 2 μl of cDNA on an ABI Prism® 7500 sequence detection system (Applied Biosystems, Foster City, CA). HuPO (human acidic ribosomal protein) primer sequences were obtained from published reports [29]. IFN-γ, SOCS1, SOCS3 and FoxP3 primer sequences were designed using Primer Express software (version 3.0, Applied Biosystems, Foster City, CA). Sequence specific primers used were

HuPO Forward 5^′^-GCTTCCTGGAGGGTGTCC-3^′^

HuPO Reverse 5^′^GGACTCGTTTGTACCCGTTG-3^′^

IFN-γ Forward 5^′^- TATGATTCTGGCTAAGGA-3^′^

IFN-γ Reverse 5^′^-CCCCAATGGTACAGGTTTCT-3^′^

SOCS1 Forward 5^′^-TTTTTCGCCCTTAGCGTGA-3^′^

SOCS1 Reverse 5′-AGCAGCTCGAAGAGGCAGTC-3^′^

SOCS3 Forward 5^′^-TGAGCGCGGCTACAGCTT-3^′^

SOCS3 Reverse 5^′^-TCCTTAATGTCACGCACGATTT-3^′^

FoxP3 Forward 5^′^-CACCTGGCTGGGAAAATGG-3^′^

FoxP3 Reverse 5^′^-GGAGCCCTTGTCGGATGAT-3^′^

Two-fold dilutions of cDNA samples were amplified to control amplification efficiency and to determine the optimal concentration required for each primer pair. HuPO was used as a control gene to calculate the ΔC_t_ values for individual samples. The relative amount of cytokine/ HuPO transcripts was calculated using the 2^-[ΔΔCt]^ method as described [30]. These values were then used to calculate the relative expression of cytokine mRNA in each of the samples tested.

### Measurement of Th1/Th2/Th17 cytokines

Cellular supernatants were collected for cytokine measurements 18 hours post stimulation, spun to collect cellular debris and stored at -70°C until tested. Concentrations of IFN-γ, TNF-α, IL-2, IL-4, IL-6, IL-10 and IL-17 were measured in cell supernatants with a Th1/Th2/Th17 Human Cytokine Flow Cytometric Bead Array kit (CBA) from BD Biosciences Ca, USA as described previously [31].

### Statistical analysis

Data is depicted as median values for each group with the IQR (inter quartile range 25^th^ to 75^th^ percentile) indicated in each case. Comparison of non-parametric data between the groups was performed using the Mann–Whitney U, Kruskal Wallis and Wilcoxon Rank non-parametric tests. Analysis was performed and data plotted using GraphPad PRISM Version 5 (GraphPad Software, San Diego, CA, USA).

## Results

### Differential *M*. *tuberculosis*-induced SOCS1 in patients with pulmonary and extra-pulmonary tuberculosis

**Figure 1:**
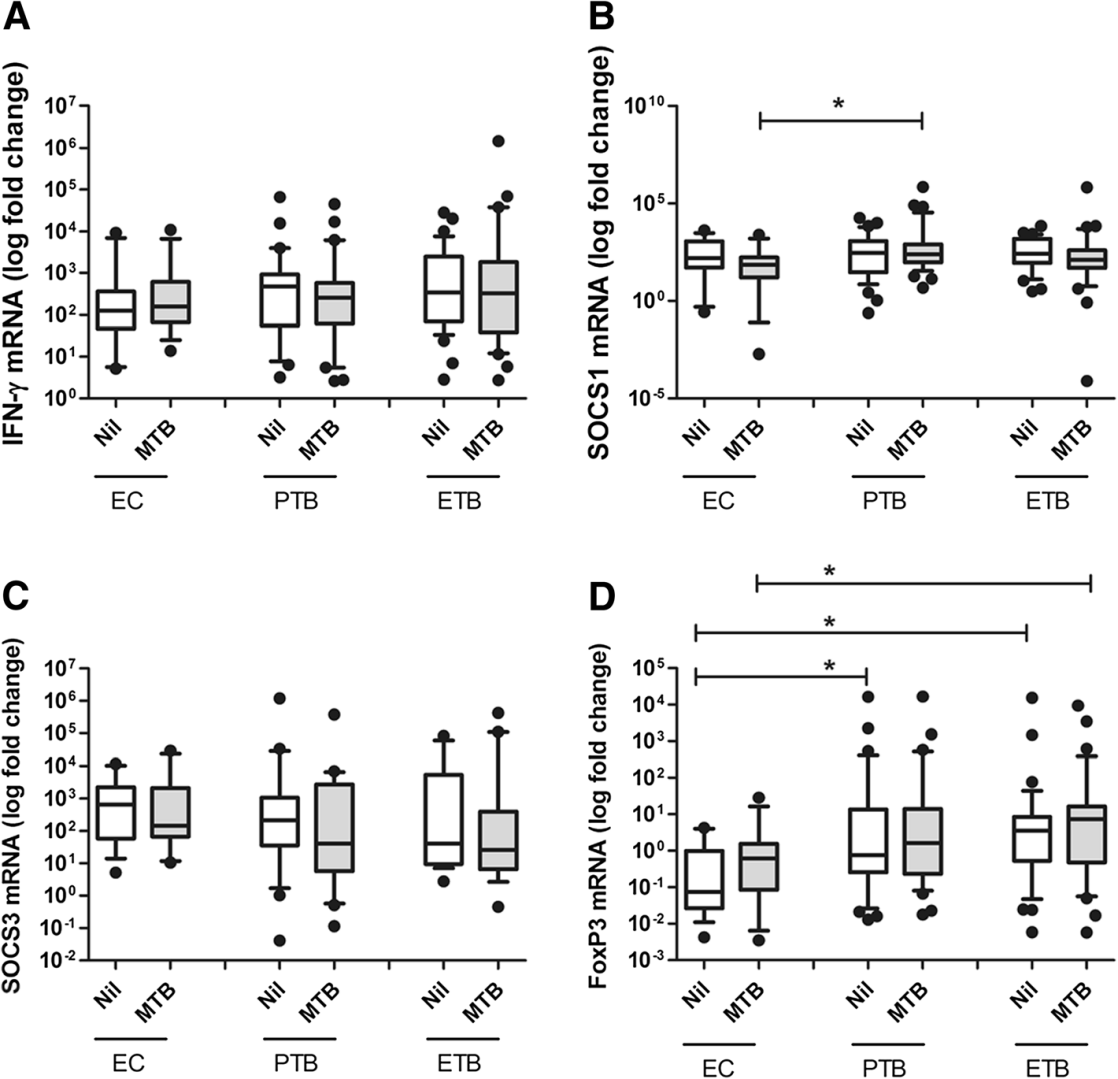
**Differential SOCS1 and FoxP3 gene expression in PTB and ETB.** PBMCs from endemic controls (EC, n = 15) and TB patients classified into pulmonary TB (PTB, n = 33) and extra-pulmonary TB (ETB, n = 33) patients were stimulated with *M*. *tuberculosis* H37Rv at MOI-2.5 for 18 hours. Total cellular RNA was harvested and subjected to RT-PCR. mRNA expression levels of IFN-γ, SOCS1, SOCS3 and FoxP3 genes from individual subjects were determined. Box and whiskers plots indicate data between 10^th^- 90^th^ percentiles with median values of each group indicated by a horizontal line. Data shows (**A**) IFN-γ, (**B**) SOCS1, (**C**) SOCS3 and (**D**) FoxP3 mRNA expression in PBMCs. ‘*’ denotes significant differences (p ≤ 0.05) as compared with EC using Mann–Whitney U non-parametric test.

We first compared the mRNA expression levels of IFN-γ, SOCS1, SOCS3, and FoxP3 in peripheral blood cells from patients with PTB, ETB and EC in either un-stimulated cells or after stimulation with *M*. *tuberculosis*. IFN-γ mRNA expression levels in un-stimulated and *M*. *tuberculosis* stimulated PBMCs isolated from EC, PTB and ETB groups were similar (Figure 1A). SOCS1 mRNA levels were comparable in un-stimulated cells from EC, PTB and ETB groups. However, *M*. *tuberculosis* -stimulated SOCS1 mRNA titers differed between EC, PTB and ETB groups (p = 0.019, using Kruskal-Wallis ‘KW’ analysis). Furthermore, *M*. *tuberculosis* -induced SOCS1 mRNA titers were higher in PTB as compared with EC (Mann Whitney-U test ‘MWU’ p = 0.0067, Figure 1B).

SOCS3 mRNA levels were comparable between EC, PTB and ETB groups in un-stimulated and *M*. *tuberculosis*-stimulated PBMCs (Figure 1C). However, we observed that FoxP3 mRNA expression levels differed between un-stimulated PBMCs from EC, PTB and ETB (KW p = 0.0035), whereby FoxP3 levels were raised in PTB (MWU p = 0.014), and ETB (MWU p < 0.001) as compared with ECs. In *M*. *tuberculosis*- stimulated cells, FoxP3 expression levels were significantly higher in ETB (MWU p = 0.021) as compared with EC, but not in the case of PTB (Figure 1D).

The direct effect of *M*. *tuberculosis* – stimulation was studied by comparing mRNA expression titers for each gene in un-stimulated and *M*. *tuberculosis*-stimulated peripheral blood cells using the Wilcoxon rank test but it was observed that there was no difference in the titers of IFN-γ, SOCS1, SOCS3 or FoxP3. This may be due to the already raised mRNA titers in TB patients as a consequence of endogenous stimulation in the *M*. *tuberculosis* infected host.

### Altered IFN-γ, IL-6 and IL-10 levels in tuberculosis

To further investigate the cytokine secretion profile in PTB and ETB cases as compared with EC we measured IFN-γ, TNF-α, IL-2, IL-4, IL-6, IL-10 and IL-17 in supernatants of un-stimulated and *M*. *tuberculosis* – stimulated PBMCs. IFN-γ, TNF-α, IL-2, IL-4 and IL-17 levels in supernatants of un-stimulated cells from TB and EC groups were similar (Table 2), whereas IL-6 levels were raised in PTB as compared with EC (MWU p = 0.018). IL-10 levels were raised in PTB (MWU, p = 0.013) and ETB (MWU, p = 0.003) as compared with EC.

**Table 2:** Increased TNFα, IL-6 and IL-10 with down-regulated IFN-γ in tuberculosis along with disease spectrum

	**Group**	**Un-stimulated**	**P value**	***M. tuberculosis *stimulated**	**P value**	**Δ MTB**	**P value**
	**Mean ± SD**	**Mean ± SD**		**Mean ± SD**		**Mean ± SD**	
**IFN-γ**	EC	0.09 ± 0.3	NS	73.61 ± 95.8	0.281	78.786 ± 97.1	NS
	PTB	0 ± 0		18.889 ± 45.3		18.621 ± 45.4	
	ETB	0.83 ± 3.9		56.70 ±173.8		57.522 ± 176.4	
**TNFα**	EC	108.20 ± 134.7	NS	2541.29 ± 2877.2	0.573	2605.19 ± 2855.91	NS
	PTB	535.81 ± 1082.35		2860.30 ± 3076.9		2378.4 ± 3146.84	
	ETB	340.02 ± 358.92		2056.48 ± 2005.8		1696.79 ± 2045.4	
**IL-2**	EC	0.109 ± 0.4	NS	24.15 ± 53.9	0.249	24.045 ± 53.92	NS
	PTB	0.148 ± 0.51		23.18 ± 99.28		21.507 ± 97.6	
	ETB	0.098 ± 0.404		36.87 ± 143.21		31.84 ± 134.92	
**IL-4**	EC	0 ± 0	NS	0 ± 0	0.762	0 ± 0	NS
	PTB	0.083 ± 0.42		0.039 ± 0.2		0.37 ± 0.19	
	ETB	0.375 ± 0.212		0.049 ± 0.288		0.049 ± 0.29	
**IL-6**	EC	651.13 ± 1018.2	0.054	10804.35 ± 1301.5	0.215	11041.6 ± 9963.4	**0.02**
	PTB	6968.3 ± 8592.02*		11782.32 ± 9677.6		7125.8 ± 9578.9	
	ETB	4677.9 ±5490.6		6581.54 ± 4757.47		2642.01 ± 3864.3*	
**IL-10**	EC	0.29 ± 1.1	**0.011**	84.78 ± 168.3	0.874	90.54 ± 171.94	NS
	PTB	55.75 ± 234.4*		76.285 ± 217.01		42.758 ± 106.81	
	ETB	51.33 ±169.17*		47.85 ± 91.7		24.76 ± 73.81	
**IL-17**	EC	0 ± 0	NS	0 ± 0	1	0 ± 0	NS
	PTB	0.075 ± 0.38		0 ± 0		0 ± 0	
	ETB	0 ± 0		0 ± 0		0 ± 0	

EC, endemic control (n = 15); PTB, pulmonary TB (n = 33); ETB, extra-pulmonary TB (n = 33).

IQR, interquartile range between 25th and 75th percentile.

* Denotes significant difference (p ≤ 0.05) as compared with EC using Mann–Whitney U non-parametric test.

P value calculated using Kruskal Wallis test. NS denotes non-significant P value (p > 0.05).

Δ is calculated by substracting *M. tuberculosis* stimulated cytokine levels from background levels from un-stimulated cells.

Subsequently, we measured *M*. *tuberculosis*-stimulated cytokine secretions in each group. No differences were observed in response to *M*. *tuberculosis* in IFN-γ, TNF-α, IL-2, IL-4, IL-10 and IL-17 secretion between EC, PTB and ETB groups (Table 2). *M*. *tuberculosis*-induced IL-6 (MWU p = 0.003) was reduced in ETB as compared with EC.

### Differential *M*. *tuberculosis* induced SOCS1 expression with varying severity of tuberculosis

Since SOCS1 and FoxP3 gene expression levels differed between PTB and ETB groups we further investigated whether they associated with the clinical severity. Therefore, we compared gene expression levels between moderately advanced (PTB-mod) and far advanced (PTB-adv) PTB, less severe (L-ETB) and more severe (D-ETB) ETB cases with ECs. In un-stimulated cells, SOCS1 mRNA levels were raised in PTB-adv as compared with PTB-mod (MWU p = 0.008) and EC (p = 0.034).

FoxP3 mRNA levels were significantly higher in PTB-adv (MWU p = 0.002), L-ETB (MWU p = 0.002) and D-ETB (MWU, p = 0.018) as compared with EC. There was no difference between IFN-γ and SOCS3 mRNA expression titers between un-stimulated cells of the TB and EC groups studied (Additional file 2: Table S2).

**Figure 2:**
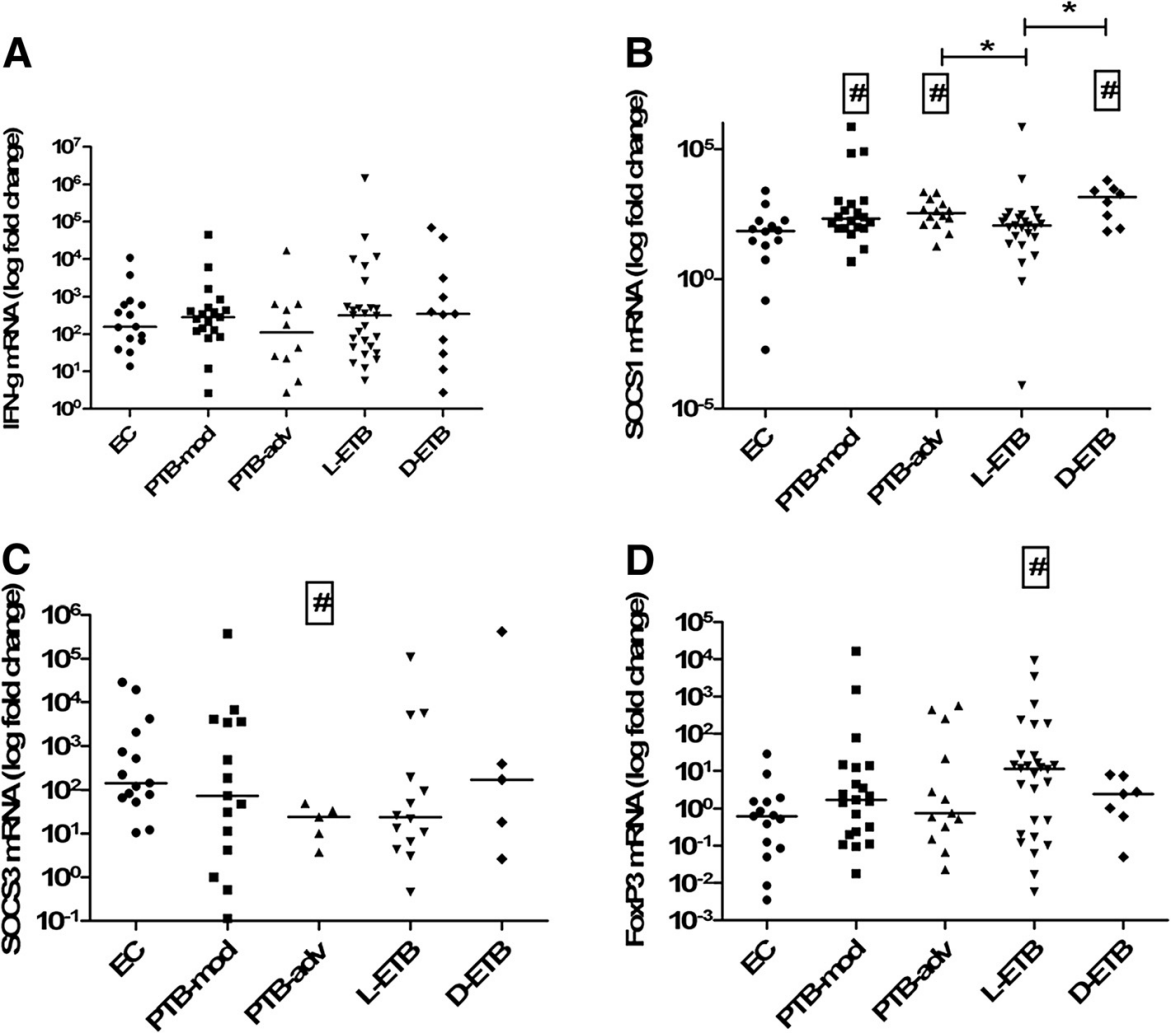
**Differential *M*. *tuberculosis *induced SOCS1 and SOCS3 expression with varying severity of tuberculosis.** PBMCs from endemic controls (EC, n = 15) and moderately advanced PTB (PTB-mod, n = 20), far advanced PTB (PTB-adv, n = 13), less severe ETB (L-ETB, n = 26) and severe ETB (D-ETB, n = 7) patients were stimulated with *M*. *tuberculosis* H37Rv at MOI-2.5 for 18 hours. Total cellular RNA was harvested and subjected to RT-PCR. mRNA expression levels of IFN-γ, SOCS1, SOCS3 and FoxP3 genes from individual subjects are depicted as scatter plots for each group with median values indicated by a horizontal line. Data shows (**A**) IFN-γ, (**B**) SOCS1, (**C**) SOCS3 and (**D**) FoxP3 mRNA expression in PBMCs. ‘#’ denotes significant differences (p ≤ 0.05) as compared with EC, ‘*’ denotes significant differences (p ≤ 0.05) between PTB-adv, L-ETB and D-ETB group using Mann–Whitney U non-parametric test.

We then compared *M*. *tuberculosis*-stimulated responses in the study subjects. IFN-γ mRNA levels did not differ between the groups studied (Figure 2A). However, *M*. *tuberculosis* induced SOCS1 mRNA gene expression levels were raised in PTB-mod (MWU, p = 0.022), PTB-adv (MWU, p = 0.014) and D-ETB (MWU, p = 0.009) groups as compared with EC (Figure 2B). Further, *M*. *tuberculosis*-stimulated SOCS1 mRNA titers were significantly greater in PTB-adv (MWU, p = 0.016) and D-ETB groups (MWU, p = 0.027) as compared with L-ETB (Figure 2B). *M*. *tuberculosis* –induced SOCS3 mRNA expression was reduced in PTB-adv (MWU, p = 0.007) as compared with EC (Figure 2C). *M*. *tuberculosis* –induced FoxP3 mRNA expression levels showed a higher accumulation in PBMCs from L-ETB (MWU, p = 0.017) as compared with EC (Figure 2D).

## Discussion

Th1 immune responses (IFN-γ, IL-12) are required for protection against infection with *M*. *tuberculosis* while a raised Th2-like cytokine profile (such as, IL-10, IL-4 and TGF-β) is implicated with disease progression in TB. To our knowledge, this is the first study to show an association between cytokine profiles in TB with the differential expression of SOCS1, SOCS3 and FoxP3 molecules in PTB and ETB. All SOCS1 and FoxP3 mRNA levels and IL-6 and IL-10 protein titers were increased in PTB. SOCS1 and FoxP3 mRNA and IL-10 protein titers were also raised in ETB. Further *M*. *tuberculosis*- stimulated moderate ETB showed decreased SOCS1 as compared with both severe ETB and far advanced PTB cases.

The raised IL-6 levels could also contribute to the increased SOCS1 expression in PTB. IL-6 might inhibit Th1 type cell responses by hampering IFN-γ, TNF-α and IL1β responses [32]. IL-10 is now recognized to be produced by almost every type of cell of the immune system, including most lymphocyte populations and cells of the innate immune system, such as antigen-presenting cells (DCs and macrophages) and granulocytes [33]. In murine models, increased IL-10 hampers protective anti- *M*. *tuberculosis* T cell responses [34]. IL-10 levels in peripheral blood cells and serum of active pulmonary TB patients were shown to be raised [35]. High IL-10 levels in both PTB and ETB were associated with an increase in FoxP3 expression. We observed FoxP3 gene expression and IL-10 secretion to be increased in both PTB and ETB compared to EC. The secretion of IL-10 by Tregs can account for the inhibition of T cell responses [36]. Neutralization of endogenous IL-10 in PBMCs from pulmonary TB patients resulted in increased T-cell proliferation and IFN-γ production [33], with enhanced proliferative responses to PPD in patients with anergic TB [37]. Thus, increased IL-10 levels in TB patients may hinder T cell responses.

*M*. *tuberculosis* –induced IL-6 and IL-10 levels in ETB were reduced as compared with EC. The reduced responsiveness of cells from *M*. *tuberculosis*-infected individuals with advanced disease is in line with previous reports which demonstrate raised endogenous levels of IL-8 and reduced *Mycobacterium*-stimulated IL8 in patients with advanced leprosy disease [38].

We measured all the cytokines simultaneously after 18 h of culture. This time point has been found to be suitable for measuring most cytokines [39] except IFN-γ responses which are increased in up to 6 days of culture [40]. Therefore it may be that we have underestimated the IFN-γ levels in our study.

All TB patients showed an increased total leukocyte but decreased lymphocyte numbers as compared with the control group. The increased SOCS1 mRNA expression observed in these patients despite a reduced lymphocyte count could be attributed to SOCS1 expression from macrophages [41,42]. We previously demonstrated that an increased SOCS1 mRNA expression associates with clinical severity in PTB patients [28]. Here we show that post *M*. *tuberculosis* stimulation, SOCS1 levels were lower in cases with less severe ETB as compared with more severe ETB and also as compared with advanced PTB. Previous studies have shown that mycobacterial antigen specific IFN-γ levels are highest in the L-ETB group [43] as compared with other sites. As SOCS1 impacts IFN-γ regulation [44], the reduced SOCS1 expression in less severe ETB group may indicate more effective T effector cell responses leading to protective granuloma formation in these cases. These data fit with previous studies that have shown higher IFN-γ responses in patients with pleural TB than those with miliary disease [9]. Also that in patients with tuberculous lymphadenitis IFN-γ responses are raised as compared to those with severe disseminated ETB [27].

*M*. *tuberculosis* stimulated SOCS3 mRNA levels were lower in PTB-adv as compared with EC. Loss of SOCS3 expression in macrophages is associated with reduced IL-12 and TNF levels [45]. Therefore, decreased SOCS3 expression in PTB-adv patients may contribute to an impaired bactericidal response to *M*. *tuberculosis*.

## Conclusions

Our results show differential expression of SOCS1 and FoxP3 together with variable IL-10 and IL-6 secretion in PTB and ETB. In addition, we observed an association between reduced SOCS1 expression and more localized ETB. These data suggest that the balance between these cytokines and SOCS1 may determine the dysregulation of immune balance in the host thereby affecting clinical severity of disease.

## Competing interests

The authors declared that they have no competing interest.

## Authors’ contributions

Conception and design: ZH and MR; Analysis and interpretation: ZH, MR, KM, RH; Drafting the manuscript for important intellectual content: ZH, MR, KM, RH. All authors read and approved the final manuscript.

## Pre-publication history

The pre-publication history for this paper can be accessed here:

http://www.biomedcentral.com/1471-2334/13/13/prepub

## Supplementary Material

Additional file 1 **Table S1.** Hematological characteristics of study subjects. Click here for file

Additional file 2 **Table S2.** Increased SOCS1 gene expression in patients with far advanced pulmonary TB. Click here for file
