# Supplementary material for: Expression of M. tuberculosis-induced suppressor of cytokine signaling (SOCS) 1, SOCS3, FoxP3 and secretion of IL-6 associates with differing clinical severity of tuberculosis
Source: BMC Infect Dis. 2013 Jan 15;13:13. doi: 10.1186/1471-2334-13-13 (PMC3562147; doi:10.1186/1471-2334-13-13)
Supplement: Additional file 1 — Table S1. Hematological characteristics of study subjects. [file 1471-2334-13-13-S1.docx]

**Table S1. Hematological characteristics of study subjects**

| **Groups** | **TLC (10e^9^/l)** | **Lymphocytes (10 e^9^/l)** | **Monocytes (10e^8^/l)** | | **Neutrophils (10e^9^/l)** |
| --- | --- | --- | --- | --- | --- |
|  | **Median (IQR)** | **Median (IQR)** | **Median (IQR)** | | **Median (IQR)** |
|  |  |  |  |  | |
| EC | 7.1 (2.7) | 33.6 (14.9) | 6.9 (2.8) | 48.3 (15.9) | |
| PTB-mod | 8.2 (2.7)* | 20.8 (12.8)* | 8.7 (4.1) | 69.6 (15.4)* | |
| PTB-adv | 10.3 (2.55)* | 17.9 (9.4)* | 3.5 (4.9) | 70.7 (8.9)* | |
| L-ETB | 8.9 (4.8)* | 22.7 (8.9)* | 6.7 (4.2) | 68 (11.2)* | |
| D-ETB | 7.4 (3.7)* | 21.5 (9)* | 7.5 (3.1) | 67 (5.7)* | |

EC, endemic control (n=15); PTB-mod, moderately advanced pulmonary TB (n=20); PTB-adv, far advanced pulmonary TB (n=13); L-ETB, less severe extra-pulmonary TB (n=26); D-ETB, severe extra-pulmonary TB (n=7); TLC, total leukocyte count; Normal ranges; TLC, (4.0-10.0 10e^9^/l); lymphocytes, (20-45 10e^9^/l); monocytes, (2-10 10e^8^/l); neutrophils, (40-75 10e^9^/l)

IQR, interquartile range between 25^th^ and 75^th^ percentile

* denotes significant difference (p ≤ 0.05) as compared with EC using Mann-Whitney U non-parametric test

**Table S2. Increased SOCS1 gene expression in patients with far advanced pulmonary TB**

| **Group** | **IFN-γ** | **SOCS1** | **SOCS3** | **FoxP3** |
| --- | --- | --- | --- | --- |
|  | Median (IQR) | Median (IQR) | Median (IQR) | Median (IQR) |
|  |  |  |  |  |
| **EC** | 123.63 (308.06) | 136.23 (530.50) | 910.09 (1920.89) | 0.27 (1.17) |
| **PTB-mod** | 474.41 (874.04) | 87.42 (508.92) | 188.64 (856.62) | 0.31 (0.73) |
| **PTB-adv** | 446.62 (1445.40) | **865.73 (1520.17)*^,#^** | 268.72 (33171.60) | **4.22 (20.19)*** |
| **L-ETB** | 254.23 (4481.23) | 215.01 (1487.66) | 104.36 (5327.88) | **3.98 (9.02)*** |
| **D-ETB** | 724.07 (1065.80) | 269.04 (1414.42) | 40.05 (61361.24) | **2.02 (5.80)*** |

EC, endemic control (n=15); PTB-mod, moderately advanced pulmonary TB (n=20); PTB-adv, far advanced pulmonary TB (n=13); L-ETB, less severe extra-pulmonary TB (n=26); D-ETB, severe extra-pulmonary TB (n=7). Data indicates mRNA expression titers from peripheral blood cells from the study groups in the absence of any stimulation. IQR, interquartile range between 25^th^ and 75^th^ percentile

* denotes significant difference (p ≤ 0.05) as compared with EC; ^#^ denotes significant difference (p ≤ 0.05) as compared with PTB-mod using Mann-Whitney U non-parametric test
