# Supplementary material for: Expression of M. tuberculosis-induced suppressor of cytokine signaling (SOCS) 1, SOCS3, FoxP3 and secretion of IL-6 associates with differing clinical severity of tuberculosis
Source: BMC Infect Dis. 2013 Jan 15;13:13. doi: 10.1186/1471-2334-13-13 (PMC3562147; doi:10.1186/1471-2334-13-13)
Supplement: Additional file 2 — Table S2. Increased SOCS1 gene expression in patients with far advanced pulmonary TB. [file 1471-2334-13-13-S2.doc]

**Table S2. Increased SOCS1 gene expression in patients with far advanced pulmonary TB**

| **Group** | **IFN-γ** | **SOCS1** | **SOCS3** | **FoxP3** |
| --- | --- | --- | --- | --- |
|  | Median (IQR) | Median (IQR) | Median (IQR) | Median (IQR) |
|  |  |  |  |  |
| **EC** | 123.63 (308.06) | 136.23 (530.50) | 910.09 (1920.89) | 0.27 (1.17) |
| **PTB-mod** | 474.41 (874.04) | 87.42 (508.92) | 188.64 (856.62) | 0.31 (0.73) |
| **PTB-adv** | 446.62 (1445.40) | **865.73 (1520.17)*^,#^** | 268.72 (33171.60) | **4.22 (20.19)*** |
| **L-ETB** | 254.23 (4481.23) | 215.01 (1487.66) | 104.36 (5327.88) | **3.98 (9.02)*** |
| **D-ETB** | 724.07 (1065.80) | 269.04 (1414.42) | 40.05 (61361.24) | **2.02 (5.80)*** |

EC, endemic control (n=15); PTB-mod, moderately advanced pulmonary TB (n=20); PTB-adv, far advanced pulmonary TB (n=13); L-ETB, less severe extra-pulmonary TB (n=26); D-ETB, severe extra-pulmonary TB (n=7). Data indicates mRNA expression titers from peripheral blood cells from the study groups in the absence of any stimulation. IQR, interquartile range between 25^th^ and 75^th^ percentile

* denotes significant difference (p ≤ 0.05) as compared with EC; ^#^ denotes significant difference (p ≤ 0.05) as compared with PTB-mod using Mann-Whitney U non-parametric test
